# The association between monocyte-to-high-density lipoprotein cholesterol ratio and type 2 diabetes mellitus: a cross-sectional study

**DOI:** 10.3389/fmed.2025.1521342

**Published:** 2025-04-28

**Authors:** Honghai Yu, Cunqing Yang, Jiao Lv, Yunyun Zhao, Guoqiang Wang, Xiuge Wang

**Affiliations:** ^1^Department of Traditional Chinese Medicine, Hunan University of Medicine General Hospital, Huaihua, China; ^2^Department of Dermatology, Guang'Anmen Hospital, China Academy of Chinese Medical Sciences, Beijing, China; ^3^Postdoctoral Research Station, Guang'Anmen Hospital, China Academy of Chinese Medical Science, Beijing, China; ^4^College of Traditional Chinese Medicine, Changchun University of Chinese Medicine, Changchun, China; ^5^Department of Endocrinology, Affiliated Hospital of Changchun University of Chinese Medicine, Changchun, China

**Keywords:** monocyte-to-high-density lipoprotein cholesterol ratio, type 2 diabetes mellitus, cross-sectional study, inflammatory biomarker, metabolic diseases

## Abstract

**Objective:**

Type 2 diabetes mellitus (T2DM) is a prevalent chronic condition often associated with low-grade inflammation. Previous studies have indicated that the monocyte-to-high-density lipoprotein cholesterol ratio (MHR) may serve as a novel inflammatory biomarker with potential predictive value for various metabolic diseases. This study aims to investigate the association between the MHR and the prevalence of T2DM in a general population, using data from the National Health and Nutrition Examination Survey (NHANES).

**Methods:**

We conducted a cross-sectional study analyzing data from five NHANES cycles spanning 2007–2016. We excluded individuals aged under 20 years, those with missing data on monocytes, HDL-C, diabetes status, or other key covariates, and extreme MHR outliers. Statistical analyses were performed using SPSS 26.0, EmpowerStats 4.1, Stata 16, and DecisionLinnc1.0. We employed weighted logistic regression models, subgroup analyses, restricted cubic splines (RCS), and threshold analyses were used to assess the MHR-T2DM association.

**Results:**

A total of 10,066 participants met the inclusion criteria, of whom 1,792 were diagnosed with T2DM. The MHR levels in the T2DM group were significantly higher than those in the non-T2DM group. After adjusting for potential confounders, elevated MHR levels were significantly associated with an increased prevalence of T2DM (*p* < 0.001, OR = 2.80, 95% CI: 1.823–4.287). Subgroup analyses revealed a significant interaction between MHR and T2DM with respect to gender (*P* for interaction < 0.05), with a stronger association in women. No significant interactions were observed for age, race, education level, poverty income ratio (PIR), body mass index (BMI), smoking status, physical activity, alcohol consumption, or hypertension (*P* for interaction > 0.05). RCS analysis indicated a significant nonlinear relationship between MHR and T2DM, with a threshold point for MHR identified at 0.51. Above this threshold, the risk of T2DM increased significantly.

**Conclusion:**

Our findings suggest that elevated MHR levels, particularly above the threshold of 0.51, are significantly associated with an increased prevalence of T2DM. The gender-specific interaction further highlights that women may be more susceptible to the impact of elevated MHR on T2DM risk. These findings suggest MHR as a potential biomarker for early T2DM screening and highlight gender-specific risk factors.

## 1 Introduction

According to the International Diabetes Federation (IDF) data from 2021, approximately 537 million individuals worldwide are living with diabetes, with 90% of these cases classified as type 2 diabetes mellitus (T2DM) (1). T2DM is a major global health challenge, significantly impairing patients’ quality of life and imposing substantial health and economic burdens. It is a leading cause of cardiovascular diseases, chronic kidney disease, neuropathy, and retinopathy, contributing to increased morbidity and mortality worldwide (2, 3). Therefore, a comprehensive understanding of its pathophysiology and the early identification of risk factors are essential for developing effective prevention and treatment strategies.

Recent studies have increasingly highlighted the critical role of inflammation in the pathogenesis of T2DM (4, 5). Monocytes, as vital components of the immune system, undergo activation and functional changes during chronic low-grade inflammation, which are closely linked to insulin resistance (6). Dysregulation of monocyte function can lead to the release of various pro-inflammatory cytokines, promoting a systemic inflammatory state that adversely affects insulin secretion from the pancreas and insulin sensitivity in peripheral tissues (7, 8). Animal model studies have provided substantial evidence of monocyte activation in diabetes. For instance, in diabetes models such as the streptozotocin (STZ)-induced diabetic mouse model, an increase in monocyte numbers and their activation is observed, accompanied by elevated levels of pro-inflammatory cytokines like TNF-α and IL-6 (9). These findings suggest that monocyte activation is a central feature of the inflammatory process in T2DM. In this context, high-density lipoprotein cholesterol (HDL-C) is recognized for its protective properties, playing a pivotal role in regulating lipid metabolism, exerting anti-inflammatory effects, and safeguarding cardiovascular health (10). Beyond its cholesterol-clearing capacity, HDL-C modulates monocyte function, thereby inhibiting their transition to an inflammatory phenotype (11, 12). Consequently, the monocyte to HDL-C ratio (MHR) emerges as a composite biomarker that may more accurately reflect an individual’s balance between inflammatory response and metabolic status.

The utility of MHR as a biomarker has been increasingly recognized in various metabolic and inflammatory conditions. For example, elevated MHR has been associated with prediabetes, a precursor state to T2DM, suggesting its potential role in early metabolic dysregulation (13). In patients with metabolic syndrome, MHR levels are significantly higher, further supporting its link to insulin resistance and systemic inflammation (14). Additionally, MHR has been implicated in the pathogenesis of non-alcoholic fatty liver disease (NAFLD), a condition often coexisting with T2DM, where it correlates with disease severity and hepatic inflammation (15, 16). Beyond metabolic disorders, MHR has also been linked to cardiovascular diseases, such as atherosclerosis, where it serves as a predictor of plaque vulnerability and adverse cardiovascular events (17). These findings underscore the dual role of MHR in both inflammation and metabolic regulation, making it a promising candidate for assessing T2DM risk. Existing studies have primarily explored MHR in the context of diabetic complications, such as vascular diseases (18). However, its potential as a biomarker for T2DM itself has not been thoroughly investigated, and this gap in knowledge warrants further research. This gap in knowledge highlights the need for further research to elucidate the relationship between MHR and T2DM, particularly in a general population setting.

Based on the existing evidence, we hypothesize that elevated MHR is independently associated with an increased prevalence of T2DM, reflecting the interplay between chronic inflammation and metabolic dysregulation. To test this hypothesis, we conducted a cross-sectional study utilizing data from the National Health and Nutrition Examination Survey (NHANES). Our study aims to systematically explore the association between MHR and T2DM prevalence while adjusting for traditional risk factors such as age, sex, body mass index (BMI), and lipid profiles. By doing so, we aim to provide new insights into the potential role of MHR as a biomarker for T2DM, offering a novel perspective on its pathogenesis and clinical implications.

## 2 Methods

### 2.1 Research population

**Figure 1:**
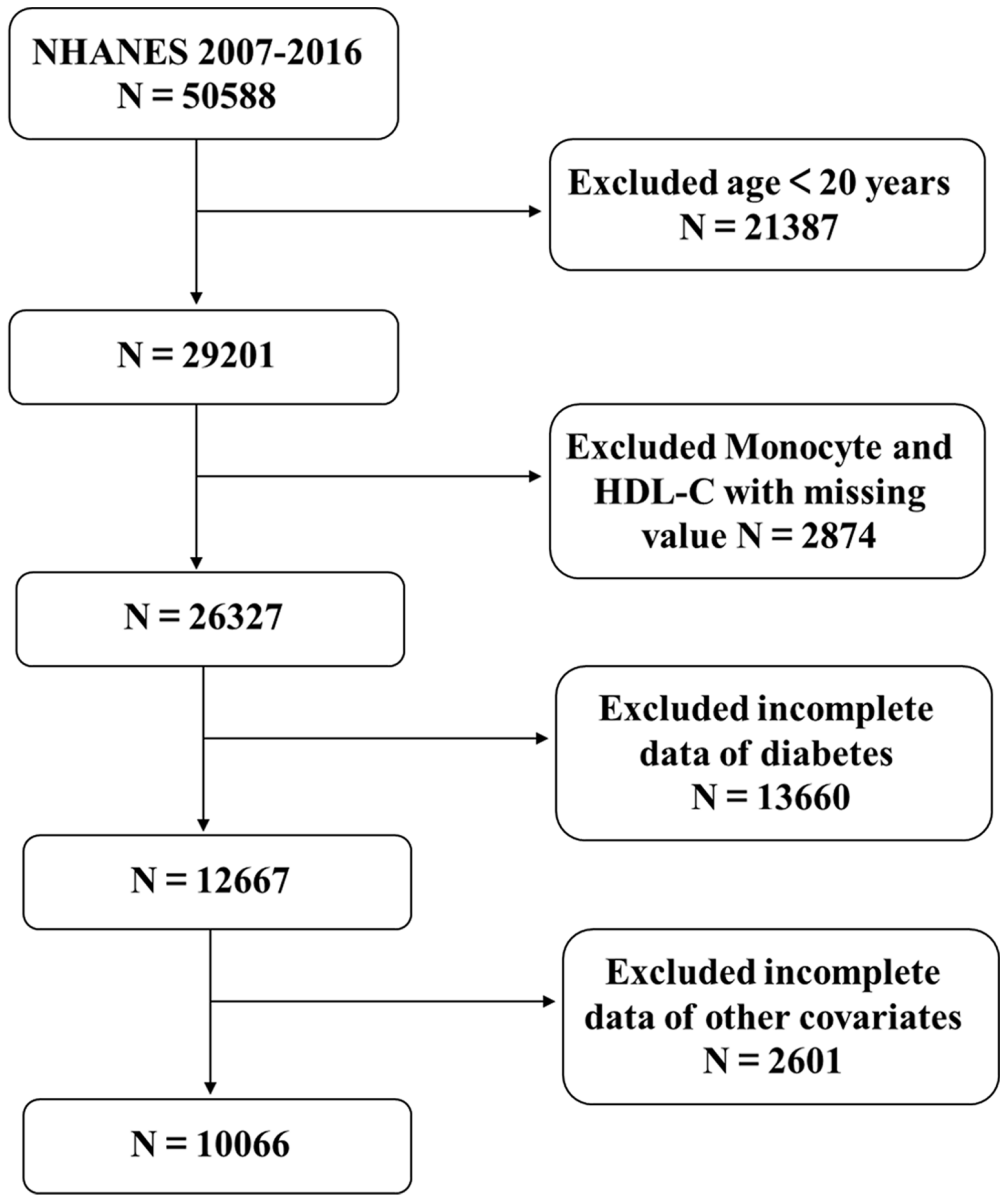
Participants and flowcharts.

The NHANES, conducted by the National Center for Health Statistics (NCHS), is a comprehensive cross-sectional study designed to ensure the representativeness of the U.S. population through a multi-stage, complex random sampling methodology. Informed consent was obtained from all participants, and the study protocol was approved by the NCHS Research Ethics Review Board.^1^ This analysis included data from five survey cycles conducted between 2007 and 2016, encompassing a total of 50,588 participants. The exclusion criteria were rigorously applied to enhance the integrity of the study: individuals aged under 20 years (*n* = 21,387); participants missing data on monocytes and HDL-C (*n* = 2,874); those lacking diabetes status information (*n* = 13,660); and individuals without complete data on education level, poverty-income ratio (PIR), alcohol consumption, smoking status, physical activity, hypertension, body mass index (BMI), total cholesterol (TCHO), alanine aminotransferase (ALT), aspartate aminotransferase (AST), and serum uric acid (SUA) (*n* = 2,591). Additionally, extreme outliers with an MHR exceeding 2 were excluded (*n* = 10). After applying these exclusion criteria, a final sample of 10,066 eligible participants was retained for analysis, as illustrated in Figure 1.

### 2.2 Exposure and outcome

Blood sample collection and processing were conducted by trained laboratory or medical technicians at NHANES. HDL-C levels were determined using either direct immunoassay or the precipitation method. The MHR was calculated by taking the ratio of the monocyte count (in 10^3^ cells/μL) to the HDL-C level (in mmol/L) (15, 19). Diagnosis of T2DM was established based on: self-report of T2DM; fasting blood glucose ≥ 7.0 mmol/L; presence of T2DM symptoms with random blood glucose ≥ 11.1 mmol/L; glycosylated hemoglobin A1c (HbA1c) ≥ 6.5% (20).

### 2.3 Covariates

Covariates included demographic information, standardized questionnaires, anthropometric measurements, and laboratory assessments. The specific covariates were as follows: age groups (20–39 years, 40–59 years, and ≥60 years), sex (male and female), racial categories (Mexican American, other Hispanic, non-Hispanic white, non-Hispanic black, and other races), education level (less than high school, high school, and above high school), and BMI, classified into three categories: normal, overweight, and obese (<25 kg/m^2^, 25–29.9 kg/m^2^, and ≥30 kg/m^2^). Smoking status was categorized as never, former, or current. Participants were asked whether they had ever smoked 100 cigarettes in their lifetime and if they currently smoke, in order to distinguish between current and former smokers. Those who reported smoking fewer than 100 cigarettes in their lifetime were classified as never smokers. Participants who were not current smokers but had previously smoked 100 cigarettes were classified as former smokers. Drinkers were defined as individuals who consumed at least 12 alcoholic beverages per year. Moreover, based on the Physical Activity Guidelines recommending ≥75 min/week of vigorous or ≥150 min/week of moderate physical activity, participants were classified into three groups: active (meeting or exceeding the recommended activity level), less active (below the recommended activity level), and inactive (no physical activity). Additionally, we included hypertension, PIR, TCHO, ALT, AST, serum creatinine (Scr), and SUA as covariates. All covariates were obtained from the NHANES database.

### 2.4 Statistical examination

Statistical analyses were performed using SPSS 26.0, EmpowerStats 4.1, Stata 16 and DecisionLinnc1.0 (21). The examinations were adjusted using weights as outlined in the NHANES guidelines, taking into consideration the 10-year data period and primary focus on blood samples. To create a weighted estimate, we referred to the “WTMEC2YR” weight variable and sampled 1-fifth of the 2-year weights from 2007 to 2016 for each individual. Categorical variables are presented as percentages, while continuous variables underwent normality testing. Data that followed a normal distribution are expressed as mean ± standard deviation, whereas data that did not conform to a normal distribution are reported as median and interquartile range to represent central tendency and dispersion. Weighted logistic regression was employed across three different models to investigate the relationship between MHR and T2DM. Model 1 was unadjusted for covariates. In Model 2, adjustments were made for age, sex, race, education level, and the PIR. Model 3 included adjustments for sex, age, race, education level, PIR, BMI, smoking status, physical activity, alcohol consumption, hypertension, TCHO, ALT, AST, Scr, and SUA. Subgroup analyses were also performed. Additionally, restricted cubic splines (RCS) were utilized to explore potential nonlinear relationships between MHR and T2DM risk. A *p*-value of <0.05 was considered statistically significant.

## 3 Results

### 3.1 Initial characteristics

A total of 10,066 participants with complete data were included in this analysis (Figure 1). Compared to the Non-T2DM group, the T2DM group was older, had a higher level of education, a greater prevalence of alcohol consumption, and reported lower levels of physical activity. Additionally, this group exhibited higher levels of Scr, SUA, MHR, and BMI. Furthermore, the T2DM group demonstrated significantly worse lipid metabolism profiles, including higher levels of TCHO and triglycerides, as well as lower levels of HDL-C. They also had elevated monocyte counts compared to the Non-T2DM group. Detailed information is provided in Table 1.

**Table 1:** Baseline characteristics of the study participants.

Parameters	Total	Non-T2DM	T2DM	*P*
*N*	10,066	8,274	1,792	
Age (%)				
20–39	3,303 (32.81)	3,196 (38.30)	134 (7.48)	<0.001
40–59	3,345 (33.23)	2,753 (33.27)	592 (33.04)	
≥60	3,418 (33.96)	2,352 (28.43)	1,066 (59.49)	
Sex (%)				
Male	4,924 (48.92)	3,963 (47.90)	961 (53.63)	<0.001
Female	5,142 (51.08)	4,311 (52.10)	831 (46.37)	
Race (%)				
Mexican American	1,496 (14.86)	1,188 (14.36)	308 (17.19)	<0.001
Other Hispanic	1,050 (10.43)	844 (10.20)	206 (11.50)	
Non-Hispanic White	4,597 (45.67)	3,892 (47.04)	705 (39.34)	
Non-Hispanic Black	1,923 (19.10)	1,490 (18.01)	433 (24.16)	
Other Race	1,000 (9.93)	860 (10.39)	140 (7.81)	
Education level (%)				
<High school	2,421 (24.05)	1,820 (22.00)	601 (33.54)	<0.001
High school	2,272 (22.57)	1,832 (22.14)	440 (24.55)	
>High school	5,373 (53.38)	4,622 (55.86)	751 (41.91)	
PIR (%)				
≤1.5	3,794 (37.69)	3,061 (37.00)	733 (40.90)	<0.001
1.5–3.5	3,222 (32.01)	2,594 (31.35)	628 (35.04)	
≥1.5	3,050 (30.30)	2,619 (31.65)	431 (24.05)	
BMI (%)				
≤25	2,967 (29.48)	2,718 (32.85)	249 (13.90)	<0.001
25–30	3,380 (33.58)	2,858 (34.54)	522 (29.13)	
≥30	3,719 (36.95)	2,698 (32.61)	1,021 (56.98)	
Smoking status (%)				
Never	5,521 (54.85)	4,622 (55.86)	899 (50.17)	<0.001
Former	2,511 (24.95)	1,912 (23.11)	599 (33.43)	
Current	2,034 (20.21)	1,740 (21.03)	294 (16.41)	
Alcohol drinker (%)				
Yes	7,269 (72.21)	6,103 (73.76)	1,166 (65.07)	<0.001
No	2,797 (27.79)	2,171 (26.24)	626 (34.93)	
Physical activity (%)				
Active	3,401 (33.79)	2,928 (35.39)	473 (26.40)	<0.001
Less active	758 (7.53)	627 (7.58)	131 (7.31)	
Inactive	5,907 (58.68)	4,719 (57.03)	1,188 (66.29)	
Hypertension (%)				
Yes	3,710 (36.86)	2,550 (30.82)	1,160 (64.73)	<0.001
No	6,356 (63.14)	5,724 (69.18)	632 (35.27)	
HDL-Cholesterol (mmol/L)	1.40 ± 0.41	1.43 ± 0.42	1.28 ± 0.38	<0.001
LDL-Cholesterol (mmol/L)	2.94 ± 0.92	2.99 ± 0.90	2.74 ± 0.97	<0.001
Monocyte number (1,000 cells/uL)	0.53 ± 0.18	0.53 ± 0.18	0.55 ± 0.19	<0.001
Triglyceride (mmol/L)	1.34 ± 0.74	1.28 ± 0.71	1.59 ± 0.81	<0.001
ALT (U/L)	25.24 ± 18.20	24.84 ± 18.29	27.08 ± 17.65	<0.001
AST (U/L)	25.86 ± 20.31	25.59 ± 19.41	27.12 ± 23.97	0.006
TCHO (mmol/L)	4.96 ± 1.05	5.00 ± 1.03	4.74 ± 1.11	<0.001
Scr (μmol/L)	79.08 ± 43.31	76.85 ± 34.66	89.40 ± 69.72	<0.001
SUA (μmol/L)	327.44 ± 83.98	322.65 ± 81.78	349.56 ± 90.26	<0.001
MHR	0.42 ± 0.20	0.40 ± 0.20	0.47 ± 0.22	<0.001

Results are expressed as mean ± standardized differences or as counts and percentages. PIR, ratio of family income to poverty; BMI, body mass index; ALT, alanine aminotransferase; AST, aspartate aminotransferase; TCHO, total cholesterol; Scr, serum creatinine; SUA, serum uric acid; MHR, monocyte-to-high-density lipoprotein cholesterol ratio.

### 3.2 The correlation between T2DM and MHR

As shown in Table 2, a significant correlation was identified between MHR and T2DM. In Model 1, no covariates were adjusted; Model 2 adjusted for demographic factors such as age, gender, race, education level, and PIR; while Model 3 included adjustments for all covariates. Overall, the analysis demonstrated a positive association between MHR and T2DM in Model 3 (OR: 2.80, 95% CI: 1.82–4.29, *p* < 0.001). Subsequent quartile analyses of MHR revealed that all models, except for the second and third quartiles in Model 3, showed statistically significant positive associations with T2DM (*p* < 0.05). In Model 3, as MHR levels increased from 0.52 to 2.00, the prevalence of T2DM correspondingly increased. Furthermore, a significant linear trend was observed in the prevalence of T2DM across MHR quartiles (P for trend < 0.001).

**Table 2:** Association between MHR quartiles and the T2DM in participants.

Model	Model 1: OR (95% CI) *p* value	Model 2: OR (95% CI) *p* value	Model 3: OR (95% CI) *p* value
MHR	5.57 (4.06–7.63) <0.001	6.72 (4.54–9.94) <0.001	2.80 (1.82–4.29) <0.001
MHR (Quartile)			
Q1 (0.04–0.27)	Reference	Reference	Reference
Q2 (0.27–0.38)	1.38 (1.11–1.73) 0.004	1.48 (1.17–1.87) 0.001	1.17 (0.92–1.48) 0.21
Q3 (0.38–0.52)	1.84 (1.49–2.29) <0.001	1.98 (1.57–2.51) <0.001	1.26 (0.98–1.62) 0.069
Q4 (0.52–2.00)	2.65 (2.15–3.26) <0.001	3.06 (2.40–3.90) <0.001	1.73 (1.33–2.24) <0.001
P for trend	<0.001	<0.001	<0.001

95% CI: 95% confidence interval; Model 1: no covariates were adjusted; Model 2: adjusted for age, sex, race, education level, PIR; Model 3: adjusted for age, sex, race, education level, PIR, BMI, smoking status, alcohol drinker, physical activity, hypertension, ALT, AST, TCHO, Scr, SUA.

### 3.3 Subgroup analysis

To ascertain the robustness of the association between MHR and T2DM across various population subgroups, subgroup analyses were conducted following Model 3. As shown in Table 3, the interaction between MHR and T2DM was statistically significant with respect to gender (*p* < 0.05). In contrast, no significant interactions were observed concerning age, race, education level, PIR, BMI, smoking status, physical activity and alcohol drinker, or hypertension (*p* > 0.05).

**Table 3:** Subgroup analysis of the association between MHR and T2DM.

Character	OR (95%CI)	*P* value	*P* for interaction
Age			0.305
20–39	4.51 (1.77–11.51)	0.002	
40–59	5.81 (2.87–11.75)	<0.001	
≥60	1.18 (0.63–2.20)	0.601	
Sex			0.002
Male	1.96 (1.13–3.39)	0.017	
Female	4.96 (2.67–9.25)	<0.001	
Race			0.761
Mexican American	2.73 (1.11–6.68)	0.028	
Other Hispanic	3.35 (1.15–9.82)	0.027	
Non-Hispanic White	3.09 (1.74–5.48)	<0.001	
Non-Hispanic Black	2.53 (1.25–5.14)	0.01	
Other Race	1.34 (0.26–7.02)	0.727	
Education level			0.056
<High school	1.90 (0.88–4.07)	0.101	
High school	3.18 (1.50–6.78)	0.003	
>High school	3.11 (1.61–6.00)	0.001	
PIR			0.704
≤1.5	2.66 (1.50–4.71)	0.001	
1.5–3.5	3.76 (1.89–7.45)	<0.001	
≥1.5	2.27 (0.90–5.72)	0.083	
BMI (kg/m^2^)			0.724
≤25	1.31 (0.55–3.14)	0.539	
25–30	2.41 (1.13–5.15)	0.023	
≥30	3.42 (1.92–6.11)	<0.001	
Smoking status			0.469
Never	2.87 (1.48–5.57)	0.002	
Former	2.51 (1.14–5.52)	0.022	
Current	2.84 (1.32–6.13)	0.008	
Alcohol drinker			0.154
Yes	3.33 (1.99–5.59)	<0.001	
No	1.51 (0.70–3.26)	0.296	
Physical activity			0.248
Active	5.72 (2.60–12.59)	<0.001	
Less active	0.68 (0.15–3.14)	0.626	
Inactive	2.67 (1.57–4.53)	<0.001	
Hypertension			0.365
Yes	2.15 (1.25–3.73)	0.006	
No	3.99 (2.15–7.39)	<0.001	

Subgroup analysis of the association between MHR and T2DM. Subgroup analysis was conducted using weighted multivariable logistic regression. BMI, body mass index; PIR, ratio of family income to poverty.

### 3.4 Nonlinear association between MHR and T2DM

**Figure 2:**
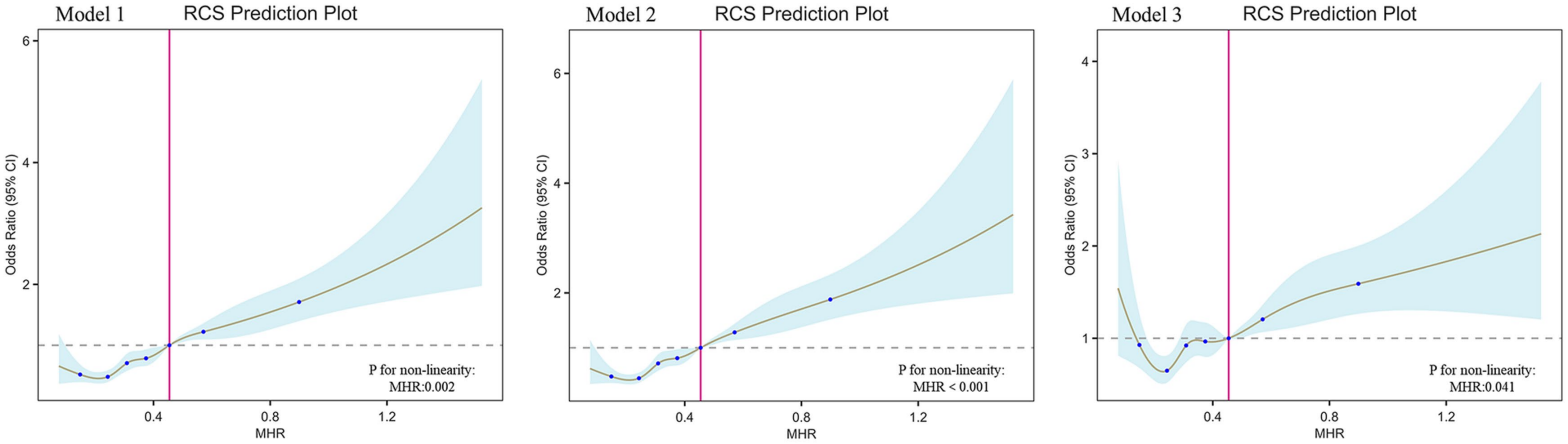
The association between MHR and T2DM. RCS shows a non-linear relationship between MHR and T2DM. The fitted regression line is a solid brown line; the black dashed line indicates the position where the OR is equal to 1; the shaded area indicates the 95% CI; MHR, monocyte-to-high-density lipoprotein cholesterol ratio.

To further elucidate the relationship between MHR and T2DM, we conducted RCS analysis, which revealed a strong nonlinear association between MHR and T2DM across all three models (*p* < 0.05 for all; Figure 2). Additionally, we performed threshold effect analysis in Model 3 (Table 4). After adjusting for covariates, the identified inflection point for MHR was 0.51. Observations indicated that when MHR is below this threshold, the risk of developing T2DM is lower (OR: 1.41, 95% CI: 0.88–2.27, *p* = 0.158). However, when MHR exceeds the inflection point, the prevalence of T2DM increases rapidly (OR: 4.47, 95% CI: 2.43–8.20, *p* < 0.001).

**Table 4:** Analysis results of the threshold relationship between MHR and T2DM.

Model 3	OR (95% CI)	*P* value
Inflection point (K)	0.51	
MHR ≤ 0.51	1.41 (0.88–2.27)	0.158
MHR>0.51	4.47 (2.43–8.17)	<0.001
Log-likelihood ratio	0.011	

Age, sex, race, education level, PIR, BMI, smoking status, alcohol drinker, physical activity, hypertension, ALT, AST, TCHO, Scr, SUA were considered in the adjustments.

## 4 Discussion

### 4.1 Summary of results

In this cross-sectional study, we analyzed data from 10,066 adult participants in the NHANES database to investigate the relationship between MHR and the prevalence of T2DM. Our findings indicate that MHR levels are significantly higher in T2DM patients compared to non-T2DM individuals, and a significant positive correlation exists between MHR and T2DM risk, even after adjusting for multiple confounding factors. RCS analysis revealed a significant nonlinear relationship between MHR and T2DM, with a threshold identified at an MHR level of 0.51. This threshold may serve as a cutoff for clinical risk stratification. Subgroup analysis further demonstrated a significant interaction between MHR and T2DM with respect to gender (*P* for interaction < 0.05). Specifically, with increasing MHR levels, women were at a greater risk of developing T2DM compared to men. This finding extends previous research by linking the gender difference in T2DM risk specifically to MHR. In contrast, no significant interactions were found concerning age, race, education level, BMI, smoking status, alcohol consumption, physical activity, or hypertension (*P* for interaction > 0.05).

### 4.2 The correlation between MHR and the increased likelihood of T2DM

Our research indicates that the MHR may be a contributing factor in the development of T2DM, aligning with previous findings. A prospective cohort study conducted in China identified the cumulative monocyte-to-high-density lipoprotein ratio (CumMHR) as an independent and stable predictor of T2DM risk (22). This study suggests that targeted assessment and management of CumMHR in low-risk populations could be a promising approach to reduce the incidence of T2DM (22). Additionally, a cross-sectional study from China demonstrated a nonlinear positive correlation between MHR and the incidence of prediabetes, further corroborating the relationship between elevated MHR levels and increased risk of prediabetes (13). The potential mechanisms linking MHR to T2DM are multifaceted, involving both monocyte-mediated inflammation and HDL-C dysfunction. Monocytes, as key mediators of the innate immune response, play a critical role in the pathogenesis of T2DM. Under conditions of chronic low-grade inflammation, monocytes are activated and release pro-inflammatory cytokines, which contribute to insulin resistance by impairing insulin signaling pathways in peripheral tissues (23–25). Experimental studies have shown that T2DM patients exhibit a fivefold increase in TLR4 expression in monocytes compared to non-diabetic controls, suggesting that enhanced inflammatory signaling in monocytes may exacerbate diabetes progression (26, 27). Animal models further support this, demonstrating that high-fat diet (HFD)-fed mice exhibit upregulated CD36 expression on monocytes, a marker of monocyte activation, which is associated with impaired glucose tolerance (28). Importantly, interventions such as low-dose aspirin (LDA) and metformin can downregulate CD36 expression and improve glucose metabolism, highlighting the therapeutic potential of targeting monocyte activation in T2DM (28). On the other hand, monocyte levels are highly dynamic and largely influenced by cholesterol metabolism disorders. One of the primary functions of HDL-C is reverse cholesterol transport, effectively clearing excess cholesterol from the body (29). Recent studies have shown that low levels of HDL-C lead to cholesterol accumulation within monocytes, enhancing the activation of inflammatory signaling pathways and resulting in more severe inflammatory responses from monocytes (30, 31). Furthermore, LDL-C has been shown to promote monocyte proliferation, while HDL-C can counteract this effect (32, 33). The relationship between T2DM and dyslipidemia is equally significant. A retrospective observational study conducted in Japan found a U-shaped relationship between HDL-C levels and clinical outcomes of T2DM, indicating that the clinical importance of HDL-C increases as glycemic status deteriorates (34). Another epidemiological study reported that for each 1 mg/dL increase in HDL-C, the prevalence of T2DM decreases by approximately 4% (35). A cohort study from the Netherlands involving 6,820 non-diabetic participants demonstrated that higher HDL-C levels are independently associated with a lower incidence of T2DM (36). Collectively, these findings reinforce the evidence linking MHR to T2DM, emphasizing its importance as a predictive factor and its potential role in the assessment and management of T2DM.

### 4.3 Analyzing the outcomes of subgroup and interaction effects

Our subgroup and interaction analyses reveal a significant finding: the likelihood of developing T2DM increases with elevated MHR levels, and this trend is more pronounced in women than in men. Previous studies support this result. A clinical study conducted in Japan found that women with dyslipidemia have a higher incidence of T2DM compared to men (37). Similarly, a cohort study in China reported a more pronounced linear increase in T2DM risk among women with dyslipidemia relative to their male counterparts (38). Notably, this gender disparity extends beyond Asian populations. A study of Italian adults demonstrated that overweight/obese women with metabolic disorders faced a disproportionately higher diabetes risk, further underscoring the potential role of sex-specific metabolic pathways (14). However, the underlying mechanisms remain unclear and are believed to involve several key factors. Firstly, women of reproductive age typically exhibit better insulin sensitivity due to the protective effects of estrogen (39). However, as they age and estrogen levels decline, the risk of developing T2DM increases (40, 41). Moreover, estrogen is linked to lipid metabolism and is associated with monocyte levels, whose fluctuations may induce changes in MHR (42, 43). Secondly, there are gender differences in insulin resistance within adipose tissue. Research indicates that visceral abdominal fat is more strongly correlated with insulin resistance in women than in men, suggesting that excess visceral fat in women is more closely associated with diabetes risk (44, 45).

In other subgroup analyses, we found that the association between MHR and T2DM was not influenced by age, race, education level, PIR, BMI, smoking status, physical activity, alcohol consumption, or hypertension. This suggests that MHR may serve as a reliable predictor of T2DM risk across diverse populations.

### 4.4 Strengths and limitations

This study utilizes data from the NHANES, which includes a substantial sample size of 10,066 participants, thereby enhancing the representativeness and reliability of the findings. Such a large-scale epidemiological study effectively captures the potential association between T2DM and the MHR within the population. MHR, which is derived from routine blood tests including monocyte count and HDL-C levels, is a simple and cost-effective biomarker that can be easily integrated into clinical practice. The identification of a specific threshold (MHR = 0.51) further enhances its utility for risk stratification, particularly in resource-limited settings. Moreover, the gender-specific association suggests that MHR could be valuable for targeted screening in women, who may benefit from early interventions to reduce T2DM risk. However, this cross-sectional study has several limitations. Some data rely on self-reported diagnoses rather than those confirmed by healthcare professionals, which may introduce bias. Although we controlled for multiple confounding factors, the influence of unknown confounders remains possible. Lastly, this study cannot establish causality between MHR and T2DM, highlighting the need for further research.

## 5 Conclusion

Our findings indicate a significant positive association between elevated MHR levels and the prevalence of T2DM. Future research should focus on longitudinal cohort studies to confirm the causal relationship between MHR and T2DM, as well as interventions targeting MHR (such as lifestyle modifications or pharmacological approaches) to prevent T2DM. Additionally, high-quality studies are needed to validate our findings and further explore the underlying mechanisms.

## Data Availability

The original contributions presented in the study are included in the article/supplementary material, further inquiries can be directed to the corresponding author.

## Footnotes

^1^ https://www.cdc.gov/nchs/nhanes/about/erb.html#print
